# Efficacy and Tolerability of a Multi‐Targeted Anti‐Acne Regimen Incorporating Salicylic Acid, Prebiotics, and Zinc PCA: A 30‐Day Clinical Trial

**DOI:** 10.1111/jocd.71034

**Published:** 2026-07-05

**Authors:** Yasmin M. Armanious, Ossama Samueal Neaz, Aya H. Abdelghany, Martina Ashraf Zaghloul, Sara Rafaat, Yostina Medhat, Nariman Ali Abdelrahim

**Affiliations:** ^1^ Research and Development Department EVA Cosmetics Company Giza Egypt

**Keywords:** acne vulgaris, dermocosmetic, lesions, prebiotics, salicylic acid, zinc PCA

## Abstract

**Introduction and Objectives:**

Acne vulgaris is a multifactorial inflammatory skin disorder that affects youths and adults, often with coincident barrier disruption and dysbiosis of the microbiome surface. The primary objective of this study was to assess the effectiveness and tolerability of a 30‐day multi‐targeted dermocosmetic regimen shown to improve acne‐prone skin while maintaining skin barrier integrity.

**Materials and Methods:**

This 30‐day clinical study was conducted on 20 volunteers with acne‐prone skin. Participants followed an 8‐product cosmetic regimen for 30 days. clinical parameters (scoring of pores visibility, seborrhoea, erythema, dryness and lesions) have been evaluated by dermatologists in conjunction with instrumental measurements of skin hydration (Corneometer CM 825) and sebum levels (Sebumeter SM 815). Subjective evaluations were collected through self‐assessment questionnaires. Statistical analyses were performed using paired t‐tests and Wilcoxon signed‐rank tests (*p* < 0.05).

**Results:**

After 30 days, there were significant decreases in sebum (−9.1%), erythema, seborrhoea, pores visibility (*p* < 0.05), and inflammatory (23%) and non‐inflammatory lesion counts (32%). Moreover, skin hydration increased (+6.2%, *p* < 0.01). Subjective assessments revealed high participant satisfaction regarding both product efficacy and skin feel.

**Conclusion:**

The dermocosmetic regimen led to significant improvements in acne‐related parameters and skin hydration; these findings support a multi‐mechanical approach towards the clinical management of acne. Further studies are recommended with a larger sample size and extended study duration, incorporating more frequent or daily monitoring to better capture treatment dynamics and improve the robustness of the findings.

## 1 Introduction

Acne vulgaris is a chronic inflammatory disease of the pilosebaceous unit and continues to be one of the most common dermatologic diseases in the world. It's the eighth most common skin disease, estimated to hit 9.4% of the world population. It affects 85% of adolescents and often continues into adulthood (particularly in females) [1]. Acne vulgaris is a non–life‐threatening yet clinically significant chronic, relapsing dermatosis characterized by visible lesions, post‐inflammatory sequelae, impaired skin barrier function, and psychosocial burden. It significantly impairs quality of life, contributing to embarrassment, anxiety, depressive symptoms, and social withdrawal. These impacts are commonly assessed using validated instruments such as the Cardiff Acne Disability Index [2, 3].

Acne vulgaris is a multifactorial disorder characterized by increased sebum production, follicular hyperkeratinization, and colonization by *Cutibacterium acnes*, alongside pilosebaceous microbiome dysbiosis and genetic, hormonal, and environmental factors, collectively driving cutaneous inflammation. It is characterized by the appearance of microcomedone, a primary lesion formed by hyperkeratotic corneocyte plugs. Closed comedones (whiteheads) result from keratin and sebum accumulation, which may progress to open comedones (blackheads). Inflammatory lesions, including papules and pustules, arise from *Cutibacterium acnes‐driven* immune activation. Follicular rupture can further release keratin, bacteria, and pro‐inflammatory lipids into the dermis, leading to deeper nodular lesions [4, 5].

Topical acne treatment is chosen based upon severity and skin tolerance. Retinoids normalize follicular epithelial desquamation and have anti‐inflammatory properties, as such they are useful for cases of mild to severe acne [4]. Benzoyl peroxide is bactericidal and leads to peeling of follicular epithelium. Clindamycin as a topical antibiotic provides antimicrobial and anti‐inflammatory properties that are useful for mild to moderate inflammatory acne [6]. Azelaic acid has several combined mechanisms of action, including antibacterial and keratinization‐normalizing effects along with anti‐inflammatory activity, making it effective in treating mild‐to‐moderate acne [7]. Salicylic acid has comedolytic (stimulating the breakdown of comedones) and anti‐inflammatory effects, mainly in mild or comedonal acne [8]. Sulfur is an antibacterial and keratolytic for treatment of mild to moderate acne and rosacea in combination with sodium sulfacetamide. Last but not the least, clascoterone: a topical antiandrogen that inhibits androgen receptors and thus diminishing sebum production as well as inflammation, appropriate for moderate to severe acne [9]. However, there are numerous drawbacks associated with their use. Often, these treatments disrupt skin barrier function which is associated with increased transepidermal water loss and decrease in lipid content which leads to dryness, irritation and tolerance issues. Moreover, non‐specific antimicrobial strategies might be among the factors responsible for skin microbiome changes and might aggravate dysbiosis instead of restoring ecological homeostasis. The prolonged use of antibiotics further raises concerns regarding the development of resistant strains of *Cutibacterium* acne. These disadvantages underscore the need for alternatives that maintain clinical effectiveness, while improving tolerability and maintaining skin barrier integrity [10, 11, 12].

The current formulation strategy utilizes a skin‐compatible, multi‐targeted approach to achieve treatment efficacy while preserving barrier function and microbiome homeostasis. Instead of using just one aggressive antimicrobial or very irritating modality, this system uses keratolytic, sebum‐regulating, and microbiome‐supportive ingredients delivered across a sequential regimen. With salicylic acid offering gradual shed to prevent follicular occlusion, niacinamide and zinc PCA work in concert with one another to regulate sebum production and reduce conditions favorable to acne‐associated microorganisms. Concurrently, the incorporation of prebiotic components (i.e., inulin and alpha‐glucan oligosaccharide) assists in stimulating the growth of beneficial skin microbiota and administering ecological balance. Tolerability is also improved using anti‐inflammatory and barrier‐supportive agents including panthenol and allantoin to reduce irritation and promote skin repair. This strategy allows sustained activity with less cumulative irritation via the distribution of these functional roles between successive product steps, which offers a more balanced and better tolerated approach for long‐term management of acne [13, 14].

This study evaluated a 30‐day anti‐acne cosmetic regimen in 20 volunteers with acne‐prone skin, made by a facial wash, scrub, corrective care, sheet mask, concealer, corrector, toner and facial wipes, using clinical scoring, instrumental measurements, and self‐assessment questionnaire. The protocol assessed skin hydration using Corneometer CM 825 and sebum using Sebumeter SM 815 at baseline and after treatment, alongside clinical changes in pores visibility, seborrhoea, erythema, dryness, and acne lesion counts.

## 2 Methods

### 2.1 Study Design

The study was designed to assess the anti‐acne regimen effectiveness and safety. The study assessed skin hydration using Corneometer CM 825 and sebum using Sebumeter SM 815, alongside clinical changes in pores visibility, seborrhoea, erythema, dryness, and acne lesion counts. In addition, at the end of the period of use, the volunteers expressed their judgment on the performance and tolerability of the treatment by filling in a self‐assessment questionnaire. At baseline, clinical and instrumental evaluations were recorded, and the participants then used the full acne‐prone skin cosmetic regimen according to the sponsor's instructions for 30 days. Assessments were conducted in a temperature‐controlled room maintained at 24°C ± 2°C. Participants were instructed to wash their face at least 3 h before evaluation and to avoid applying any facial products for at least 12 h before baseline measurements.

### 2.2 Participants

Twenty volunteers were enrolled in the trial, comprising 5 males and 15 females, with a mean age of 28.2 years, all of whom were in generally good health and had acne‐prone skin. Eligibility criteria required Caucasian subjects aged 15–50 years who were able to comply with study instructions, attend the follow‐up visit, complete the informed consent process, and avoid UV exposure and tanning beds throughout the study period. The participants received instructions to avoid using any other facial products on the tested areas throughout the entire duration of the research study.

Exclusion criteria included pregnancy or lactation, use of topical or systemic medications that could influence the results of the study, history of unusual skin reactions to cosmetic or toiletry products or known sensitivity to any component of the tested formulations, presence of systemic diseases or skin disorders such as eczema or psoriasis that could affect evaluation or increase subject risk, use of anti‐acne treatments within 30 days before enrollment, and participation in another clinical investigation with comparable objectives within the same 30‐day period. Subjects who withdrew by personal choice, developed intercurrent illness, or experienced irritant or allergic reactions related to the treatment were considered dropouts.

### 2.3 Test Products and Use Regimen

The tested skin care program included eight cosmetic products which were designed for acne‐prone skin, as shown in Table 1. The study participants followed the sponsor's product application guidelines for a period of 30 days. The regimen included daily application of corrective care and toner, alternating use of facial wash, scrub, and wipes, periodic use of the sheet mask, and local application of concealer/corrector as needed.

**TABLE 1:** Eva skin clinic anti‐acne skin care program.

Product name	Mode of usage	Ingredients	Frequency	Route of administration
Fresh Restart Facial Wash	Apply a small amount on the hands, lather and massage into wet face using gentle circular moves, then rinse well.	Salicylic Acid, Niacinamide, Glycerin, Propylene Glycol, Panthenol, Inulin, Zinc PCA, Alpha‐Glucan Oligosaccharide.	Three times a week alternative to scrub or wipes	Topical
Exfoliating Legend Facial Scrub	Apply a small amount to a dampened face, then massage gently for one minute. Rinse off thoroughly.	Pumice, Propylene Glycol, Glycerin, Salicylic Acid, Zinc PCA, Polyquaternium‐11, Niacinamide, Panthenol, Inulin, Alpha‐Glucan Oligosaccharide, Coconut Acid, Allantoin, Caprylyl Glycol.	Twice a week alternative to face wash or wipes	Topical
Break‐out Solution Corrective Care	Apply a thin layer on affected areas and massage gently using fingertips.	Salicylic Acid, Propylene Glycol, C12‐15 Alkyl Benzoate, Cetyl Alcohol, Dimethicone, Inulin, Zinc PCA, Glyceryl Stearate, Niacinamide, Panthenol, Alpha‐Glucan Oligosaccharide, Allantoin, Sorbitan Isostearate.	Twice a day for 30 days	Topical
Skin Retreat Sheet Mask	Place it on a cleansed face. Leave the mask from 15 to 30 min, then remove it. Massage remaining product gently onto face and neck.	Alpha‐Glucan Oligosaccharide, Glycerin, Panthenol, Niacinamide, Allantoin, Inulin, Zinc PCA, *Hamamelis Virginiana* (Witch Hazel) Leaf Water.	Once or twice a week	Topical
Confidently Covered Corrector + Concealer (medium beige and green)	Cleanse face and neck. Tap green color corrector gently on localized breakouts, then apply concealer and blend smoothly using fingertips.	Salicylic Acid, Pentaerythrityl Tetraisostearate, Dimethicone, Propylene Glycol, Octyldodecanol, Dicaprylyl Carbonate, Isononyl Isononanoate, Talc, Panthenol, Inulin, Niacinamide, Zinc PCA, Xylose, Hydrogenated Lecithin, Sorbitan Isostearate, Alpha‐glucan Oligosaccharide.	Daily as needed.	Topical
Balancing Genius Toner	Apply to clean face and neck using a cotton pad in an upward motion. Do not rinse.	Salicylic Acid, Propylene Glycol, Inulin, Niacinamide, Zinc PCA, Panthenol, Alpha‐Glucan Oligosaccharide, *Hamamelis Virginiana* (Witch Hazel) Leaf Water, Hexyl Cinnamal, Benzyl Salicylate.	Daily after the face wash/scrub/wipes	Topical
Cleansing Rescue Facial Wipes	Gently wipe face and neck to remove impurities and traces of makeup.	Niacinamide, Propylene Glycol, Inulin, Panthenol, Glycerin, *Hamamelis Virginiana* (Witch Hazel) Leaf Water, Zinc PCA, Niacinamide, *Aloe Barbadensis* Leaf Extract, Alpha‐Glucan Oligosaccharide, Tocopheryl Acetate.	Twice a week as alternative to face wash and scrub.	Topical

### 2.4 Clinical Assessment

Clinical evaluation was performed by visual examination at baseline and after 30 days. The parameters scored were pores visibility, seborrhoea, erythema, and dryness using a 4‐point ordinal scale (0 = absent, 1 = mild, 2 = moderate, 3 = severe). Acne lesions were counted by typology and classified as non‐inflammatory lesions (open comedones and closed comedones) and inflammatory lesions (papules and pustules) [15].

### 2.5 Instrumental Assessment

Skin hydration was measured using Corneometer CM 825, which assesses stratum corneum hydration. The instrument is based on the physical principle of capacitance. The device consists of a square‐shaped sensor (area: 49 mm^2^) with a special glass covering the front, on a mobile axis, connected to the base unit by a spiral cable. When the front surface of the sensor is pressed against the skin, a number appears on the liquid crystals display. This value is directly proportional to the amount of water contained in stratum corneum and represents the hydration level of cutaneous surface, expressed by corneometric units (c.u.), which are the instrument arbitrary units. The measurement must be made on a flat area, taking care to use constant pressure for not too long time, to avoid inducing occlusion phenomena with the probe. The device can measure values ranging between 0 and 150 corneometric units [16]. Skin sebum was measured using Sebumeter SM 815, which determines surface lipid content and reports results in micrograms per square centimeter. The device is fitted with a manual counter featuring a ribbon 0.1 cm wide and with a surface contact area of 64 mm^2^, used to absorb the sebum from the skin. After setting the counter to zero, the head of the device is held against the skin for 30 s, then inserted inside the optical device. When the head carrying the ribbon surface which has soaked up the sebum is inserted in the device, a closed circuit is created which lights a beam projected towards the ribbon. This beam is reflected by a mirror onto a photocell and amplified by a micro‐ammeter. The greater the quantity of lipids present on the ribbon, the more transparent the film will become: the numerical values read on the display are directly proportional to the transparency of the ribbon and hence to the quantity of lipids present on it. Sebometric values are expressed in micrograms/cm2. Measurements were performed on one randomized half of the face to minimize site bias [17].

### 2.6 Subjective Assessment

At the end of the 30‐day period, participants completed a self‐assessment questionnaire evaluating cleansing efficacy, perceived gentleness, exfoliating and dermopurifying performance, blemish concealment, mattifying effect, sebum‐normalizing efficacy, hydration, softness, and overall satisfaction. Participants also reported any adverse or secondary effects such as redness, burning, itching, dryness, or desquamation.

### 2.7 Statistical Analysis

Descriptive statistics were calculated as mean values, standard deviations, and variations. Normality was assessed using the Kolmogorov–Smirnov test [18]. Instrumental data were compared between baseline (*T*_0_) and day 30 (*T*_30_) using a paired *t*‐test for dependent and parametric data [16], while clinical ordinal data were analyzed using the Wilcoxon signed‐rank test for dependent and non‐parametric data. Statistical significance was set at *p* < 0.05 [17].

## 3 Results

### 3.1 Clinical Assessment

After 30 days of product use, clinical assessments revealed statistically significant improvements. As shown in Table 2, mean scores for pores visibility decreased from 2.2 (±0.7) to 1.9 (±0.7) (*p* < 0.05), seborrhoea decreased from 2.2 (±0.9) to 1.6 (±0.9) (*p* < 0.01), erythema decreased from 1.8 (±0.9) to 1.1 (±1.0) (*p* < 0.01), and dryness decreased from 0.7 (±1.0) to 0.3 (±0.6) (*p* < 0.05). Furthermore, the lesion count analysis (Tables 3 and 4) showed a statistically significant reduction in the total number of both non‐inflammatory lesions (from 9.8 ± 9.0 to 8.6 ± 7.8, *p* < 0.05) and inflammatory lesions (from 7.7 ± 10.5 to 5.8 ± 7.6, *p* < 0.05). Acne lesions were counted by typology and classified as non‐inflammatory lesions (open comedones and closed comedones) and inflammatory lesions (papules and pustules). As indicated in Tables 3 and 4, there are statistically significant decreases in the sum of acne lesions of both non‐inflammatory lesions (from 9.8 ± 9.0 to 8.6 ± 7.8, *p* < 0.05) and inflammatory lesions (from 7.7 ± 10.5 to 5.8 ± 7.6, *p* < 0.05) evidenced after 30 days of treatment. The treatment induced statistically significant decreases in the level of all the observed clinical parameters.

**TABLE 2:** Mean values, standard deviations, variations and statistical significance (Wilcoxon test).

Clinical parameters	*T*_0_	*T*_30 days_	*p*
Pores visibility	2.2 ± 0.7	1.9 ± 0.7	*p* < 0.05
Seborrhoea	2.2 ± 0.9	1.6 ± 0.9	*p* < 0.01
Erythema	1.8 ± 0.9	1.1 ± 1.0	*p* < 0.01
Dryness	0.7 ± 1.0	0.3 ± 0.6	*p* < 0.05

**TABLE 3:** Mean values and standard deviations.

Acne lesions		*T*_0_	*T*_30 days_
Non‐inflammatory lesions	N° open comedones	5.4 ± 7.5	5.2 ± 5.8
	N° closed comedones	4.4 ± 3.9	3.4 ± 3.3
	N° papules	4.8 ± 5.1	4.1 ± 4.2
Inflammatory lesion	N° pustules	2.9 ± 5.8	1.8 ± 3.9

**TABLE 4:** Mean values, standard deviations and statistical significance (Wilcoxon test).

	*T*_0_	*T*_30 days_	*p*
Sum of non‐inflammatory lesions	9.8 ± 9.0	8.6 ± 7.8	*p* < 0.05
Sum of inflammatory lesions	7.7 ± 10.5	5.8 ± 7.6	*p* < 0.05

### 3.2 Instrumental Assessment

**FIGURE 1:**
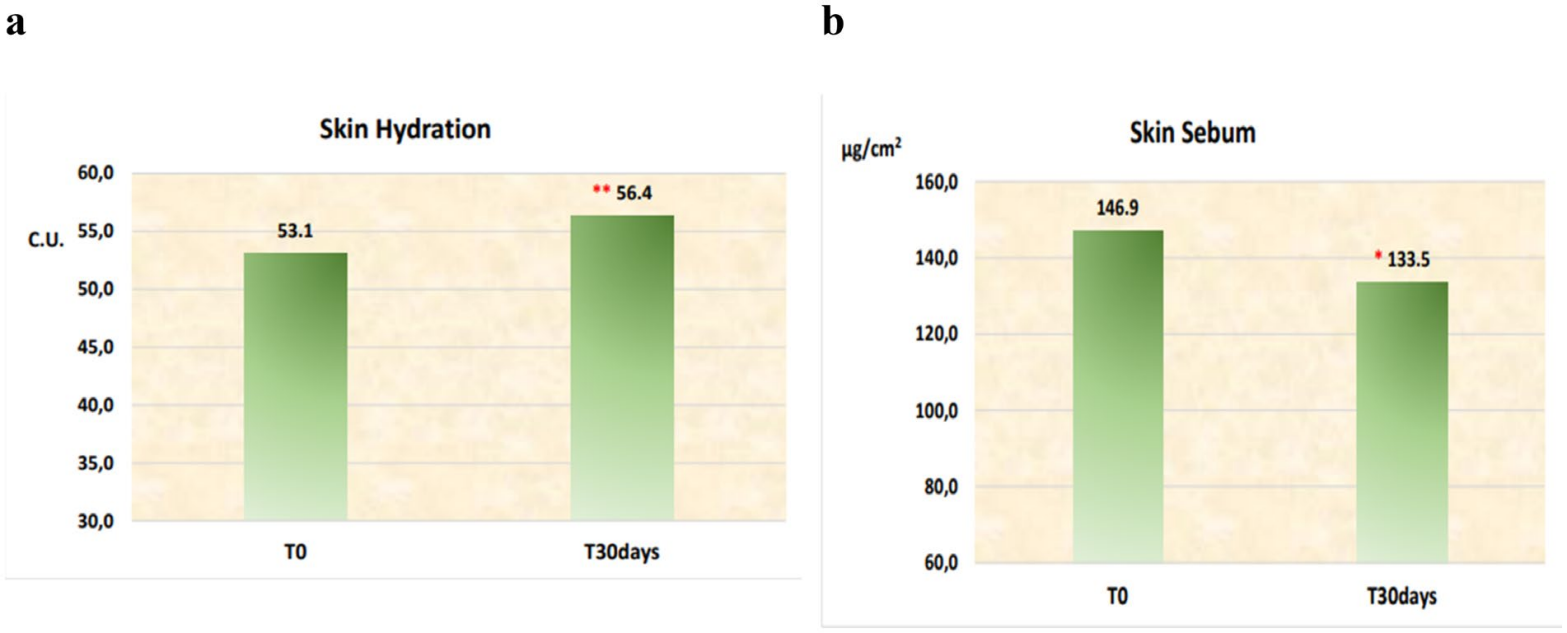
(a) skin hydration mean values recorded before and after 30 days of treatment (**p < 0.01). (b) skin sebum mean values recorded before and after 30 days of treatment (*p < 0.05).

Instrumental measurements supported the clinical findings. As represented in Table 5 and Figure 1a, skin hydration, measured with Corneometer, increased significantly from a mean baseline value of 53.1 (±8.5) to 56.4 (±9.6) after 30 days, corresponding to a +6.2% variation after 30 days of treatment. Conversely, as shown in Table 6 and Figure 1b, skin sebum levels, measured with Sebumeter, decreased significantly from 146.9 (±36.6) to 133.5 (±46.2), representing a −9.1% reduction (*p* < 0.05).

**TABLE 5:** Mean values, standard deviations, variation and statistical significance (*t*‐test) for skin hydration.

*T*_0_	*T*_30 days_	Variations (%) *T*_30 days_‐*T*_0_	*p* *T*_0_ versus *T*_30_ days
Mean 53.1	mean 56.4	3.3	*p* < 0.01
st. dev. 8.5	st. dev. 9.6	(6.2%)	

**TABLE 6:** Mean values, standard deviations, variation, and statistical significance (*t*‐test) for skin sebum.

*T*_0_	*T*_30 days_	Variations (%) *T*_30 days_‐*T*_0_	*p* *T*_0_ versus *T*_30 days_
Mean 146.9	Mean 133.5	−13.4	*p* < 0.05
st. dev. 36.6	st. dev. 46.2	(−9.1%)	

### 3.3 Subjective Evaluation

**FIGURE 2:**
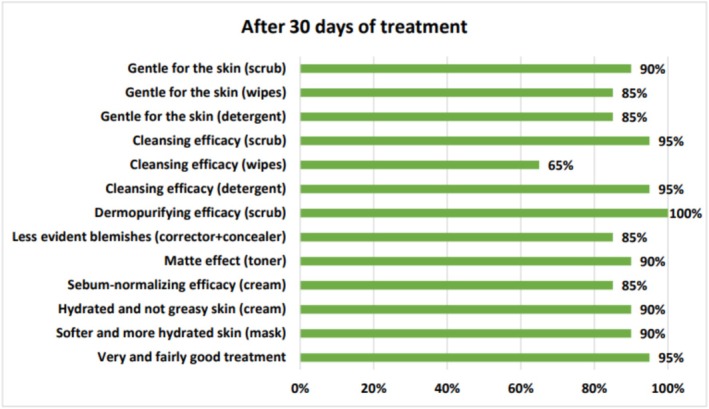
Subjective evaluation results of the volunteers about the performances of the products after 30 days of use.

Participant self‐assessments after 30 days of treatment showed high efficacy across multiple attributes. The scrub was reported as gentle for the skin by 90% of participants, while wipes and detergent were both rated at 85%. Cleansing efficacy was highest for the scrub and detergent at 95% each, compared to 65% for wipes. Notably, dermopurifying efficacy of the scrub reached 100%. Less evident blemishes (corrector + concealer) were reported by 85%, and the toner provided a matte effect for 90%. Sebum‐normalizing efficacy of the cream was rated at 85%, while 90% found the cream to leave skin hydrated and not greasy. The mask left skin softer and more hydrated for 90% of participants. Overall, 95% rated the treatment as very good or fairly good (Figure 2). Despite these positive outcomes, one subject (Subject #19) experienced intense redness, desquamation, and burning after the scrub and detergent use. In addition, other 5 subjects experienced mild to moderate desquamation and dryness after the use of scrub, toner, wipes, and detergent.

## 4 Discussion

This 30‐day clinical study was conducted on 20 volunteers with acne‐prone skin. Clinical parameters (scoring of pores visibility, seborrhoea, erythema, dryness and lesions) have been evaluated by dermatologists in conjunction with instrumental measurements of skin hydration and sebum levels. Subjective evaluations were collected through self‐assessment questionnaires.

This study showed a statistically significant reduction compared to baseline in sebum production, lesion counts, erythema, and dryness, and an increase in skin hydration with the use of a 30‐day multi‐targeted dermocosmetic regimen. These results mirror the emerging notion for acne management that balancing efficacy with barrier preservation may offer best therapy through multi‐mechanistic approaches [18].

The observed clinical improvements are attributed to the complementary and synergistic actions of the active ingredients. Salicylic acid contributes to follicular desquamation and inhibiting microcomedone formation, resulting in reductions in both inflammatory and noninflammatory lesions [4]. This approach showed that niacinamide and zinc PCA regulate sebum production in accordance with the Sebumeter reductions observed; niacinamide was also shown to enhance ceramide synthesis and epidermal barrier function, which most likely explains the simultaneous increase in Corneometer‐measured hydration even though keratolytic agents were used [13, 14].

In addition, the incorporation of prebiotic components (inulin and α‐glucan oligosaccharide) introduces a microbiome‐supportive dimension to the regimen. Increasing evidence suggests that acne is associated with microbial imbalance and that maintaining microbial diversity may improve clinical outcomes [9, 10]. Prebiotics are designed to restore microbial homeostasis while maintaining barrier integrity as broad‐spectrum antimicrobials (e.g., benzoyl peroxide or clindamycin) may alter skin ecology and contribute to irritation [9, 10, 11]. This is supported by randomized clinical data showing that combined products with salicylic acid, niacinamide, and zinc with additional microbiome‐targeting ingredients can achieve higher efficacy than those with 5% benzoyl peroxide with better tolerability profiles [23].

The addition of panthenol and allantoin had barrier supportive and anti‐irritant effects, in line with their previously reported soothing and reparative properties [13]. In contrast to studies reporting barrier impairment with keratolytic‐based regimens, the present findings suggest that appropriate formulation design can preserve or even enhance skin hydration.

A distinctive feature of this regimen is its integration of an active‐ingredient‐infused concealer and color corrector within the same 8‐product system. Unlike conventional coverage products that offer only cosmetic camouflage, these formulations are designed to deliver continuous sebum‐regulating and anti‐inflammatory benefits while concealing blemishes, which addresses both the physiological and psychosocial aspects of acne. This dual functionality may improve adherence by reducing the number of separate products a consumer needs to purchase and apply, and it ensures that even the ‟coverage” step contributes to treatment, not just concealment.

Despite these positive outcomes, one subject (Subject #19) experienced intense redness, desquamation, and burning after the scrub and detergent use. In addition, other 5 subjects experienced mild to moderate desquamation and dryness after the use of scrub, toner, wipes, and detergent. These findings highlight that tolerability is not solely dependent on active ingredients but is critically influenced by regimen architecture, including product type, application frequency, and cumulative skin stress.

Several limitations should be acknowledged. Among the limitations, the small sample size (*n* = 20) and lack of a control group limit the generalizability of the results, in addition to the short duration, which may not be truly reflective of long‐term efficacy. Additionally, the absence of objective assessments of transepidermal water loss and microbiome composition limits mechanistic insight.

## 5 Conclusion

The current study shows that the use of a multi‐targeted dermocosmetic treatment protocol can effectively improve the skin condition in patients suffering from acne in a period of 30 days. The effect was noted via decreasing sebum production, lesion counts, erythema, and improving skin hydration. Nevertheless, considering limitations such as small study sample size and short study period, it may be stated that the above mentioned dermocosmetic approach can prove to be very effective in treating mild‐to‐moderate acne cases while preserving skin barrier function.

## Author Contributions

M.A.Z., S.R., and Y.M. were responsible for conceptualization, formulation, methodology, and questionnaires, N.A.A. and A.H.A. wrote and edited the original manuscript draft. O.S.N. was involved in the supervision, resources and reviewing the final manuscript. Y.M.A. was involved in conceptualization, supervision, and resources.

## Funding

The authors have nothing to report.

## Ethics Statement

This non‐interventional study did not require approval from an ethics committee prior to its start. The current study was carried out in compliance with the quality assurance system requirements at LabAnalysis Group (https://www.labanalysis.it) in Casanova Lonati, Italy. The study was conducted according to Declaration of Helsinki principles in the framework of the laboratory's quality system.

## Consent

All participants were informed about the nature, purpose, and possible risks of the study before enrollment and provided written informed consent prior to participation.

## Conflicts of Interest

The authors declare no conflicts of interest.

## Data Availability

The data that support the findings of this study are available from the corresponding author upon reasonable request.
